# Effect of L-Glutamic Acid on the Composition and Morphology of Nanostructured Calcium Phosphate as Biomaterial

**DOI:** 10.3390/ma16031262

**Published:** 2023-02-01

**Authors:** Fatah Takabait, Sergio Martínez-Martínez, Laila Mahtout, Zahra Graba, Pedro J. Sánchez-Soto, Luis Pérez-Villarejo

**Affiliations:** 1 Laboratoire de Technologie des Matériaux et de Génie des Procédés (LTMGP), Faculté des Sciences Exactes, Université A. Mira-Béjaïa, Terga Ouzemmour, Béjaïa 06000, Algeria; 2 Department of Chemical, Environmental and Materials Engineering, Higher Polytechnic School of Linares, University of Jaén, Campus Científico y Tecnológico, Cinturón Sur s/n, 23700 Linares, Spain; 3 Institute of Materials Science of Sevilla (ICMS), Joint Center of the Spanish National Research Council (CSIC), University of Sevilla, Isla de la Cartuja, 41092 Seville, Spain

**Keywords:** calcium phosphate, L-glutamic acid, phosphoric acid, brushite, apatite, chelate complexes, biomaterials

## Abstract

Calcium phosphate (CaP) with several chemical compositions and morphologies was prepared by precipitation using aqueous solutions of L-Glutamic acid (H_2_G) and calcium hydroxide, both mixed together with an aqueous solution (0.15 M) of phosphoric acid. Plate-shaped dicalcium phosphate dihydrate (brushite) particles were obtained and identified at a lower concentration of the solution of the reactants. The Ca/P ratio deduced by EDS was ~1, as expected. The nanoscale dimension of carbonate apatite and amorphous calcium phosphate, with variable Ca/P ratios, were revealed by X-ray diffraction (XRD) and scanning electron microscopy and energy dispersive X-ray spectroscopy analysis (SEM-EDS). They were characterized in medium and high concentrations of calcium hydroxide (0.15 M and 0.20 M). The equilibria involved in all the reactions in aqueous solution were determined. The thermodynamic calculations showed a decrease in the amount of chelate complexes with an increase in pH, being the opposite of [CaPO_4_^−^] and [CaHG^+^]. This fluctuation showed an evident influence on the morphology and polymorphism of CaP particles obtained under the present experimental conditions, with potential use as a biomaterial.

## 1. Introduction

Biomaterials based on calcium phosphates are widely used in the biomedical field, especially for bone defect replacement and teeth implants. They have raised great research interest due to their wide range of applications and their biological properties (biocompatibility, bioactivity, and biodegradability), as well as their compositional similarity to bone and teeth mineral [1,2,3,4]. Compared to stoichiometric hydroxyapatite (HAp), apatite usually has a molar ratio of calcium to phosphate of less than 1.67 and is very prone to various ion substitution, such as Mg^2+^, Na^+^, Sr^2+^, CO_3_^2−^, etc. Carbonate is the most abundant substitution in apatite. Its content can vary in the range of 3 to 8 wt.% and a substitution relating to OH-/PO_4_^3−^ ions can occur in position A and/or B, respectively. These impurities increase its bioactive properties and solubility [5,6,7]. The term “apatite” involves calcium phosphate with Ca/P molar ratios within 1.5–1.67 [2]. However, calcium hydrogen phosphate dihydrate with the stoichiometry CaHPO_4_·2H_2_O, a mineral phase known as brushite, has a Ca/P molar ratio equal to 1. It is generally in a stable phase in weakly acidic environments. It is, therefore, considered to be in a metastable phase in the formation of less soluble apatite and octacalcium phosphate (OCP) [8,9,10].

Many pioneering works reported synthetic methods of acquiring calcium phosphate (CaP) [5,6,11,12]. Among these methods are chemical precipitation, sol-gel processing, mechanochemical synthesis, microwave decomposition, microemulsion technology, sonochemical techniques, hydrothermal synthesis, and others [1,2,3,4,5,6,13]. Each method leads to the formation of CaP crystals with variable structural and morphological properties and, consequently, of different purity, size, and crystallinity, as well as intrinsic polymorphism [5]. In addition to the method and synthetic conditions, the control of CaP precipitation can be inspired by biomineralization processes. A wide range of organic additives, including biomacromolecules, have been explored [14]. Gómez-Morales et al. [6] reported that citrate and non-collagenous proteins play a key role in the transition from amorphous calcium phosphate (ACP) to apatite, and also, in the thickness and crystal orientation of apatite on the collagen fibrils. First, an amorphous precursor is formed in this process. Then, it is transformed into a crystalline phase while maintaining its morphology. Furuichi et al. [15] prepared brushite in gelatin. These authors showed that nanofibrous hydroxyapatite (HAp) is formed by the dehydration of dicalcium phosphate dihydrate (DCPD) when applied as a biomaterial. Our previous studies on the synthesis of submicron and nanosized vaterite particles, with potential interest as biomaterials, showed the effect of an organic additive on this process [16,17].

Glutamic and aspartic acids are amino acids (AAs). They are highly present in the extracellular matrix of biologically calcified tissues. Further, they used in vitro to synthesize HAp-AAs composites with reduced crystal dimension and to promote osteoblast proliferation [18,19]. Composites brushite-L-Glutamic were synthesized in a mixed solvent and water (Ethanol-H_2_O) at a molar ratio of 20:60 [18,19,20]. These results were interpreted as the fact that pH strongly affects the precipitation of calcium phosphates and the change in the ionization of the functional group AAs [20].

An interesting early review focused on the previous studies of CaP for biomedical applications, giving a description of the processes in biomaterials [2]. CaP is considered the most widely used inorganic biomaterial in applications of bone tissue engineering, such as medical implants. This is attributed to its satisfactory biocompatibility, bioactivity, non-toxicity, similarity with the inorganic phase present in the bone, and osteoconduction characteristics [21]. The main phases of CaP for applications in the design of implants with different properties and performances are biphasic calcium phosphates, tricalcium phosphates, and HAp [22]. For instance, progress in the synthesis of high-crystallinity CaP has been the synthesis of nanoparticle composites using gold showing an antimicrobial response [23].

The CaP family of materials has been used in a variety of applications, such as bone cement, orthopedics, and coating [1,2,3,4], and is injectable and hardens by combining crystals with plate and needle forms in situ [23,24]. Cheah et al. [25] appraised the development, characterization, and biological performance of different materials obtained by synthesis with applications for bone, periodontal, and dental tissue regeneration, including CaP types of cement. This paper is a part of an interesting recent collection of research articles and reviews “describing numerical and experimental design techniques definitively aimed at improving the scaffold performance, shortening the healing time, and increasing the success rate of the scaffold implantation process”, as pointed out by Bocaccio [26]. Furthermore, it should be remarked that the use of CaP in cosmetics, as reviewed by Carella et al. [27], shows the-state-of-the-art and future perspectives. These authors described the principal synthesis parameters that influence the characteristics of CaP, as well as the formation of different crystal phases from ACP to HAp, following an increase in thermodynamic stability. These parameters are pH, temperature, solvent, time, and surfactants. In this sense, Nosrati et al. [28] studied the brushite chemical precipitation on graphene sheets in an aqueous–ethanolic medium to increase the mechanical and biological properties of CaP simultaneously. In this study, computer-assisted modeling was used to understand the processes. However, for a deep study, the knowledge of equilibrium reactions involved in these wet processes are basic and fundamental.

The objective of this study was to investigate the effect of L-Glutamic acid on the composition and morphology of CaP from calcium hydroxide and phosphoric acid in aqueous solution. Thus, interesting and novel results are reported here showing the variations observed in the chemical composition and morphology of CaP as a nanostructured material, with potential application as a biomaterial, in particular, dicalcium phosphate dihydrate (DCPD, brushite). Ca(OH)_2_ was used in aqueous solution (0.1–0.15 M), keeping the concentration of H_3_PO_4_ (0.15 M) constant and changing the concentration of L-Glutamic acid (maximum 0.1 M). The thermodynamic equilibria involved in all reactions in the aqueous solution and the amount of each ionic species were determined.

## 2. Experimental

All chemical reagents were of analytical grade, including calcium hydroxide Ca(OH)_2_, phosphoric acid (85 wt.% H_3_PO_4_), and L-Glutamic acid (thereafter glutamic acid). These chemicals were supplied by Aldrich and used as received.

The synthesis resulted in the preparation of two solutions as described in Table 1. The first one contained 100 mL of glutamic acid and calcium hydroxide at different concentrations. The second solution contained only 50 mL of phosphoric acid in an aqueous 0.15 M solution. Deionized water was used for the preparation of all aqueous solutions. These solutions, prepared as described above, were mixed together by vigorous stirring at 700 rpm for 1 h at 25 °C. White products were formed by reaction, collected by filtration, and washed repeatedly with deionized water. The solids were dried overnight at 103 °C prior to any characterization.

An X-ray diffraction (XRD) study of the powdered solids was performed using a Philips X’Pert Pro PANalytical equipment (Malvern, UK) with CuKα incident radiation (λ = 1.5418 Å), operating at 40 kV–40 mA. The diffractograms were collected between 10–80 2θ-angle with a 2θ-step size of 0.001. The software for this equipment was used for mineral phase identification and analysis. The average crystallite size of the particles was calculated by XRD patterns of the peak corresponding to (002) reflection of apatite. Scherrer´s equation [29] was used in the form:

(1) D=(Kλ)/(βcosθ)

In Equation (1), D is the average crystallite size perpendicular to the reflecting planes, K is the Scherrer constant close to unity (0.9), λ is the wavelength of incident X-rays, β is the full width of the peak at half of the maximum (FWHM), and θ is the diffraction or Bragg angle. Instrumental broadening was previously measured using a crystal standard to perform these calculations under the best conditions [29]. The estimated error in the crystallite size comes from instrumental broadening. Thus, it was evaluated to be ± 1 nm.

Attenuated total reflectance Fourier transform infrared spectroscopy (ATR-FTIR) was used to confirm the characteristic vibration bands of various CaP polymorphs. The equipment was an ATR-FTIR spectrometer fitted with an Agilent diamond ATR sample interface (Agilent Cary 630, Santa Clara, California, USA). The spectra were recorded in the region of 400–4000 cm^−1^ in absorbance mode with 128 successive scans at a resolution of 4 cm^−1^.

The evaluation of carbonate content or carbonation degree of the CaP samples was quantified by a validated method based on FTIR methodology, as described by Grunenwald et al. [7].

Finally, the morphology of the samples was studied by scanning electron microscopy (SEM), recording the electron images using Quanta SEM Philips equipment (Amsterdam, The Netherlands), coupled to energy dispersive X-ray spectroscopy (EDS) for chemical analysis. The samples were coated by the sputtering of thin gold films before being examined using SEM. The magnification used depended on the sample analyzed and varied between 8000×, 24,000×, and 30,000×. The EDS analysis was practiced for points or areas depending on the sample and the interest zone.

## 3. Results and Discussion

### 3.1. XRD Characterization of CaP Synthesized Powders

**Figure 1:**
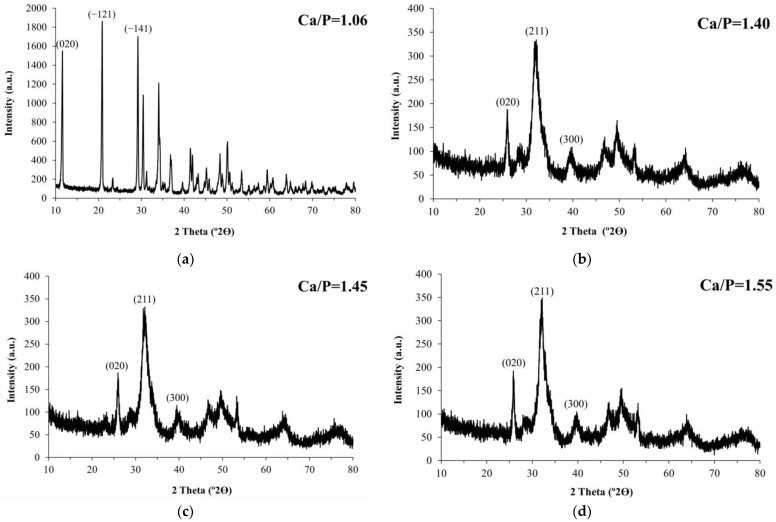

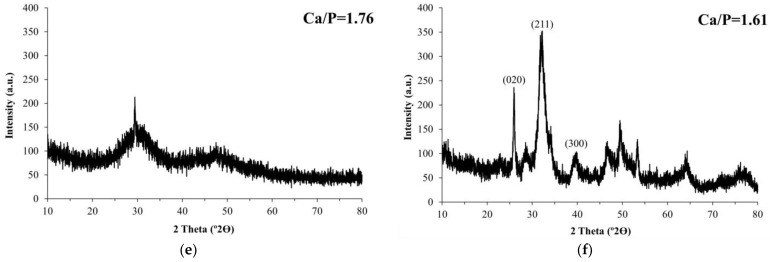
XRD patterns of the different powders synthesized in this investigation (see Table 1 for experimental conditions): (**a**) Sample A, Ca/P = 1.06; (**b**) Sample B, Ca/P = 1.40; (**c**) Sample C, Ca/P = 1.45; (**d**) Sample D, Ca/P = 1.55; (**e**) Sample E, Ca/P = 1.76; (**f**) Sample F, Ca/P = 1.61.

The XRD analysis (Figure 1) indicated that different CaP phases were obtained in the presence of glutamic acid as an additive under the experimental conditions described in Table 1, as follows: (1) dicalcium phosphate dihydrate (brushite) in sample A; (2) apatite successively in samples B, C, and D; and (3) amorphous CaP in sample E. In absence of the additive, only apatite was precipitated, corresponding to the XRD diagrams A, B, C, D, E, and F, respectively.

The characteristic peaks of brushite were only observed in the first experiment (Figure 1a), with high intensity for (020), (−121), and (−141) reflections, indicating the formation of DCPD with good crystallinity at pH = 4.18 [8,30]. This fact was indeed confirmed by chemical analysis by EDS of brushite crystals with a plate-like shape, as observed by SEM, and an average Ca/P molar ratio of 1.06.

Furthermore, the high-intensity peaks at °2θ = 11.6 and 20.1, as observed in Figure 1, can be attributed to the *β* form of L-Glutamic acid [31]. This result suggested the possible formation of a DCDP-L-Glutamic acid composite. However, there were no characteristic peaks (Figure 1e) corresponding to CaP crystalline phases in the experiment conducted for the synthesis of sample E (Table 1). The presence of amorphous CaP can be evidenced in accordance with the previous findings of Ikawa et al. [20].

The formation of apatite in other samples (Figure 1b–d,f) can be evidenced mainly by the diffraction lines located at °2θ = 25.85 and broad X-ray peaks at °2θ = 31.8 and 32.8, being assigned to (002), (211), and (300) reflections, respectively. This broad band in the diffraction pattern is indicative of the precipitation of poorly crystalline HAp nanoparticles [5,6,20,32]. The crystallite size along the c-direction of the apatite powders was calculated from (002) reflection using equation 1 [29]. The results showed that the crystallite size decreased slightly from ~27 ± 1 nm for samples B and C to ~24 ± 1 nm for sample D. It is an indication that the increase in pH in the presence of glutamic acid solution reduces the crystallite size of apatite crystal. This result agrees with previous reports suggesting that glutamic acid inhibits crystal growth [19].

### 3.2. FTIR Characterization of CaP Synthesized Powders

**Figure 2:**
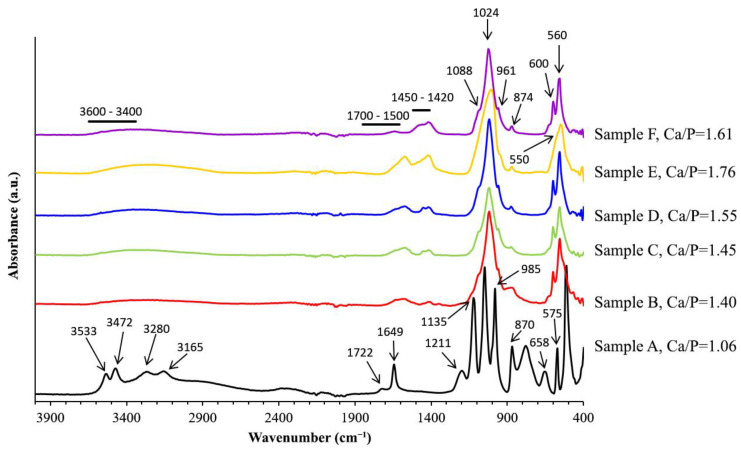
ATR-FTIR spectra of CaP powders A–F synthesized according to the present experimental conditions (Table 1): Sample A, Ca/P = 1.06 (black line); Sample B, Ca/P = 1.40 (red line); Sample C, Ca/P = 1.45 (green line); Sample D, Ca/P = 1.55 (blue line); Sample E, Ca/P = 1.76 (yellow line); Sample F, Ca/P = 1.61 (pink line).

Figure 2 summarizes all the ATR-FTIR spectra of CaP powders synthesized according to the experimental conditions described in Table 1. The results confirmed the polymorphs revealed by the previous XRD study, such as DCPD (Figure 2, sample A), with the identification of two doublets at 3543 and 3587 cm^−1^ and 3280 and 3165 cm^−1^ attributed to the O-H stretching of water molecules, while the vibrational mode present at 1649, 658 cm^−1^, and 1722 cm^−1^ [9,33]. The absorptions at 1135, 1059, 985, 870, 575, and 525 cm^−1^ were attributed to the PO_4_^3−^ group in brushite. The bands appearing at 1211 cm^−1^ and 874 cm^−1^ were assigned to in-plane P-O-H bending and P-O(H) stretching, respectively [32,34]. Additional absorption peaks of amino acid in the region 2800–3200 cm^−1^ were observed (N-H and C-H stretching bands of NH_3_^+^ and CH_2_ groups, respectively) [9,20,34].

The IR bands characteristic of apatite were identified in the following four samples: sample B, sample C, sample D, and sample F, with a notable variation in their intensity (Figure 2). The bands characteristic of PO_4_^3−^ groups (1088, 1024, 961, and 600, 560 cm^−1^) and OH^−^ were distinguished at 628 cm^−1^. Other bands that appeared at 3568, 3436, and 1650 cm^−1^ were also attributed to adsorbed water molecules in the samples [5,18,32,35].

The IR bands at 1450, 1420, and 874 cm^−1^ revealed the presence of CO_3_^2−^ groups replacing the phosphate position or B-type substitution [5,36]. Thus, carbonate adsorbed from atmospheric carbon dioxide was confirmed by FTIR analysis of these samples. The quantitative evaluation of carbonate content, or carbonation degree in apatite (bio)minerals, was performed according to the FTIR methodology proposed and validated by Grunenwald et al. [7]. The results indicated 1.79 ± 0.5, 2.04 ± 0.5, and 4.86 ± 0.5% for samples D, E, and F, respectively. These results resemble the amount of carbonate in the bone nano apatite [5]. In addition to bands related to amino acids as indicated above, the relative intensity of the band corresponding to AAs groups in the range of 1500 to 1700 cm^−1^ increased with the increase in the pH, in agreement with a previous report [37]. The IR spectrum of amorphous calcium phosphate (Figure 2, sample E) presents some bands previously discussed above and others different, mainly the broad bands observed at 550 and 1070 cm^−1^ assigned to phosphate groups [38]. The carbonation degree, estimated by the same validated method of Grunenwald et al. [7], indicated a value of ~4.24 ± 0.5% for sample E. Comparing all the values of carbonate content, sample F contained the highest value, and sample D the sample had the lowest value.

### 3.3. SEM Characterization of Synthesized CaP Powders

**Figure 3:**
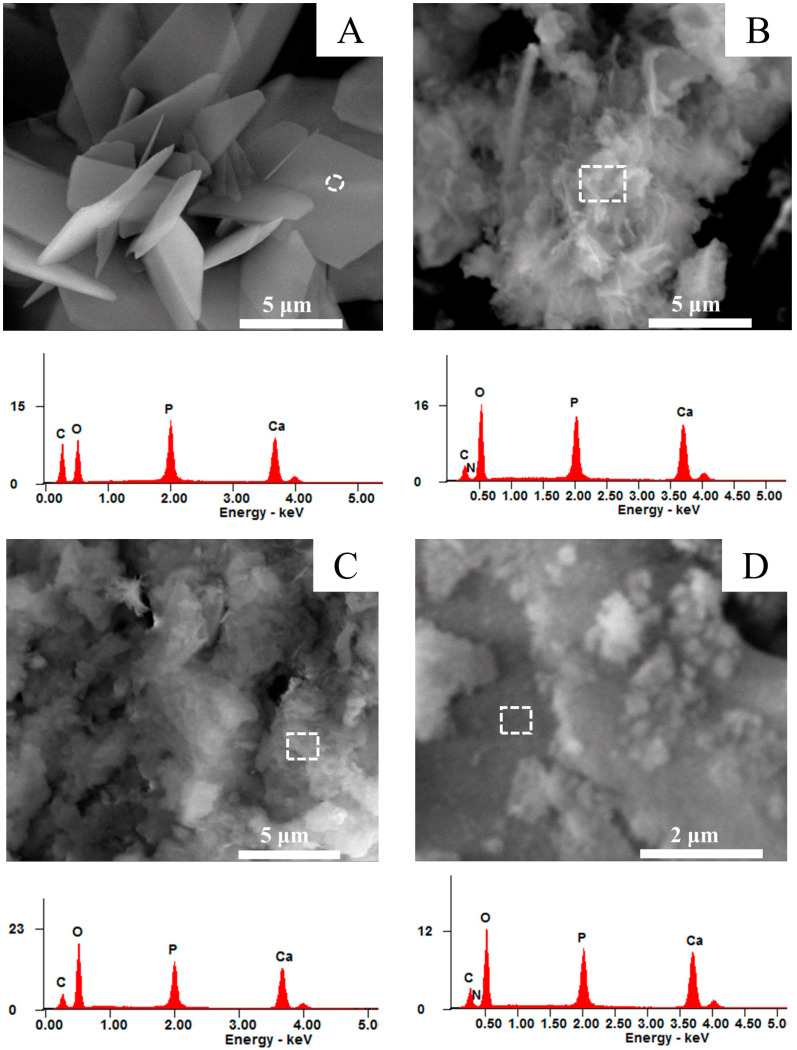

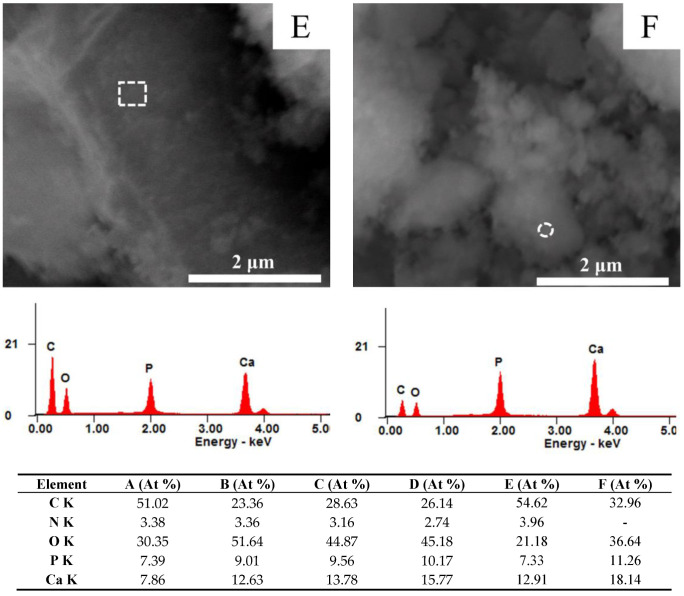
SEM images and EDS spectra of CaP samples: (**A**) Sample A, Ca/P = 1.06 (8000×); (**B**) Sample B, Ca/P = 1.40 (8000×); (**C**) Sample C, Ca/P = 1.45 (8000×); (**D**) Sample D, Ca/P = 1.55 (24,000×); (**E**) Sample E, Ca/P = 1.76 (30,000×); (**F**) Sample F, Ca/P = 1.61 (30,000×). It is included the atomic percentages of the elements C, N, O, P and Ca determined by EDS analysis in these samples.

SEM micrographs (Figure 3) revealed that the synthesized CaP powders at low pH (4.18) exhibited a well-defined plate-shaped morphology, with particle sizes in the range of 1 to 5 µm. This arrangement may be associated with the influence of amino acids on the growth of DCPD along preferential directions, which was similar to previous reports [5,39].

The morphologies of the particles formed under other pH conditions (Table 1) were unclear in the SEM micrographs (Figure 3B–F). It is an indication of the nanoscale dimension of the powder synthesized under the present experimental conditions showing agglomerated particles. They were typical of poorly crystalline carbonated apatite and amorphous calcium phosphate [40].

The EDS spectra are presented in Figure 3. In the case of Figure 3A,F, the EDS analyses are point analyses (circle), while Figure 3B–E correspond to analyses of the entire marked area (rectangle). It can be observed that the atomic Ca/P ratio deduced by EDS for sample A was ~1 (the exact value is 1.06), as expected for pure brushite. For the rest of the samples, atomic Ca/P ratios were 1.40, 1.44, 1.55, and 1.61, for samples B, C, D, and F, respectively. These values represent carbonate/apatite which is lower than that of the stoichiometric hydroxyapatite (value is 1.67) [5,6].

Finally, an interesting result appeared in the present experiments: glutamic acid was adsorbed on the CaP formed as precipitated white products. The important relative proportions of the C, O, and N atoms in the corresponding EDS spectra (Figure 3B–D) revealed it. The atomic Ca/P ratio for sample E, calculated from the results of EDS analysis, was 1.76. This value revealed that amorphous calcium phosphate containing glutamic acid complexed with the excess amount of calcium ions was possibly present in the aqueous medium. It would be a matter for additional study to determine the adsorption of glutamic acid in solution under isothermal conditions.

### 3.4. Ion Concentrations in Reaction Solutions

All the chemical equilibriums and concentrations of ionic species in the supersaturated solutions (Table 1) calculated from their corresponding dissociation constants must be considered, as shown in Table 2. It included ions pair CaH_2_PO_4_^+^ and CaPO_4_^−^. Exclusively the chelates consisting of calcium ions bonded with glutamic acid (CaG, CaHG-), which assumed that only 1:1 metal chelates formed. As an approximation, the amount of CO_3_^2−^ ions was ignored in the following calculations with respect to each of the calcium phosphate phases.

All activity coefficients of ions were calculated from the modified Debye–Hückel equation proposed by Davies [45], as follows:

log γ_i_ = 0.5211Z^2^[√µ/(1 + √µ) − 0.3µ] (2)

and µ is the ionic strength of the solution, given by the equation:

µ = ½ Σ_i_C_i_Z^2^ (3)

where C_i_ and Z are the molar concentration and charge of the ion, respectively.

The results of the theoretical analysis of each typical experiments are summarized in Table 3. All results were in good agreement with the phase diagram for the system Ca(OH)_2_-H_3_PO_4_-H_2_O [5]. Ion pairs showed that they evolved with high concentrations in order relative to the pH and their charge. The acidic pH produced positively dominant ion pairs such as CaH_2_PO_4_^+^ and CaHG^+^. However, at basic pH, the negatively charged ions thrived at high concentrations such as PO_4_^−^. It is known that many factors govern and guide crystal formation and growth, as discussed previously. The influence of the initial concentration of Ca(OH)_2_ and glutamic acid on the chemical composition of the precipitates obtained can be explained by changes that occurred in the pH.

Low concentration led to a drastic decrease in pH until 4.18 (pH ≤ pK_i2_ of glutamic acid), and thus was mostly generated by conversion and incomplete dissociation of H_3_PO_4_ and H_2_G to CaH_2_PO_4_^+^ and CaHG^+^ with high concentrations (42.51, 14.37 mM, respectively), as was reported earlier [44,46].

On the contrary, Ca(OH)_2_ at this pH range decomposed completely into Ca^2+^ and OH^−^, which is reasonable by complete dissolution due to the neutralization and phase nucleation, as the acid is added and the increase in the volume of H_2_O. Therefore, dicalcium phosphate dihydrate (brushite) nucleated easily and grew (with plated shapes as observed by SEM) on the orientation along a particular direction, regulated by the molecular interaction with the CaHG^+^ complex [9], according to the reaction:

CaH_2_PO_4_^+^ + CaOH^+^ = CaHPO_4_ + H_2_O

The same experiment was carried out in the absence of glutamic acid (sample F, Table 1), and the pH suddenly increased to 10.56, Furthermore, there was a radical change in the concentration of chemical species in this reaction, including the final product, which was apatite. However, if the precursor concentration of Ca^2+^ was kept constant at 0.15 M with a gradual decrease in the concentration of glutamic acid from 0.1 to 0.05 mM in experiments B, C, and D, it allowed the same final product as apatite. However, with a pH increase and change in size, the crystallinity of the formed crystals decreased, as was reported by Tavafoghi and Cerruti [19], who studied the role of amino acids in hydroxyapatite mineralization. An interesting fact raised in this pH range (pH = 5.20–8.45) or pH between pK_2_ and pK_3_ of glutamic acid was the concentration of CaH_2_PO_4_^+^ and CaHG^+^ decreased inversely relative to the concentration of CaPO_4_^−^ and with that of other ions such as Ca^2+^, CaHPO_4_, and CaG.

The large excess of ion metals (the value is Ca^2+^ = 24.25 mM) involved in the experimental conditions (sample E) created by the highly concentrated solutions initially mixed (pH ≥ pK_i3_ of glutamic acid) led to the formation of amorphous CaP, which appeared to be only the more important phase. This is in agreement with the species that occurred in the reactions, mainly ion pairs such as CaPO_4_^−^ and CaG being much larger than that of CaHPO_4_ and CaHG^+^ in a more alkaline medium [44,47]. The stability of the complexes formed in the reaction medium prevented the nucleation of apatite and the transformation of precipitated amorphous CaP into apatite form is suppressed [20].

Additionally, as discussed above, this process was usually affected by simultaneous factors such as supersaturation, the action of glutamic acid on the pH, and electrostatic interactions that occurred with other precursors and agitation. They exercised control over the form and also the polymorph of the CaP formed in the final product. Thus, the present results are of interest as they provide information on the formation and evolution of CaP, and hence, are of relevance when the application of synthesized CaP products as biomaterials is being investigated. It will be a subject for future research.

## 4. Conclusions

In the present investigation, CaP with several chemical compositions and morphologies was prepared by precipitation using aqueous solutions with different molar ratios of glutamic acid and calcium hydroxide, mixed together with an aqueous solution (0.15 M) of phosphoric acid. The nano-sized precipitated products, carbonate apatite, and amorphous calcium phosphate, and their atomic Ca/P ratios were calculated using EDS. Quantitative determination of carbonate content was by FTIR. The interest in the synthesized material powders as biomaterials is emphasized. Dicalcium phosphate dihydrate, brushite, was precipitated in the medium under the present experimental conditions, although amorphous calcium phosphate and carbonate apatite can be obtained at the nanoscale dimension. The plate-shaped brushite particles were evidenced by SEM and confirmed by EDS, with a Ca/P ratio of ~1, as expected for pure brushite.

The equilibria involved in the reactions in aqueous solutions were investigated for a deep study of calcium phosphate synthesis. The phase, size, and morphology of the CaP precipitated were mainly influenced by the pH of the medium in the presence of glutamic acid. The thermodynamic calculations showed that the amount of chelate complexes [CaH_2_PO_4_^+^] decreased with an increase in pH, which is the opposite of [CaPO_4_^−^] and [CaHG^+^]. This fluctuation showed an evident influence on the morphology and polymorphism of CaP particles obtained under these experimental conditions. It is concluded that at pH = 10.63, calcium apatite was formed. However, at pH ≤ pK_2_ of glutamic acid, mineral brushite was precipitated. At pH > pK_3_, calcium phosphate, as an amorphous phase, was obtained. At a pH between these two pK_i_, apatite was produced.

## Figures and Tables

**Table 1:** Experimental conditions for the synthesis of calcium phosphates, showing the volumes of reactants, measured pH, Ca/P molar ratio, and the final products of the reaction.

Sample	Ca(OH)_2_	L-Glutamic Acid	H_3_PO_4_	pH Measured	Ca/P	Final Product
	V = 100 mL		V = 50 mL			
A	0.1 M	0.025 M	0.15 M	4.14	1.06	DCDP
B	0.15 M	0.1 M	0.15 M	5.17	1.40	Apatite
C	0.15 M	0.075 M	0.15 M	6.66	1.45	Apatite
D	0.15 M	0.05 M	0.15 M	8.52	1.55	Apatite
E	0.2 M	0.075 M	0.15 M	9.58	1.76	CaP Amp
F	0.15 M	0 M	0.15 M	10.63	1.61	Apatite

**Table 2:** Equations and equilibria to speciation calculations (H_2_G = L-Glutamic acid).

Reaction	Ksp (25 °C)	Ref
$\mathrm{Ca}\;(\mathrm{OH}{)}_{2}\;⇄$ CaOH^+^ + OH^−^	K _sp(289.15K)_ = 10^−3.86^	[41]
${\mathrm{CaOH}}^{+}\;\;⇄$ Ca^2+^ + OH^−^	K _sp(289.15K)_ = 1/25.12	[42]
$H_{2}G\;⇄$ G^−^ + H^+^	K _sp(289.15K)_ = 8.51 × 10^−5^	[43]
${\mathrm{HG}}^{-}\;\;\;⇄$ G^2−^ + H^+^	K_sp(289.15K)_ = 3.39 × 10^−10^	[43]
H_3_PO_4_ ⇄ H_2_PO_4_^−^ + H^+^	K _sp(289.15K)_ = 7.13 × 10^−3^	[44]
H_2_PO_4_^−^ ⇄ HPO_4_^2−^ + H^+^	K _sp(289.15K)_ = 6.31 × 10^−8^	[44]
${\mathrm{HPO}}_{4}2^{-}\;⇄$ PO_4_^3−^ + H^+^	K _sp(289.15K)_ = 4.22 × 10^−13^	[44]
$H^{+}+{\mathrm{OH}}^{-}\;⇄$ H_2_O	k_e_ = 10^−14^	
${\mathrm{Ca}}^{2+}+{\mathrm{HG}}^{-}⇄$ CaHG^+^	K_sp_ = 10^1.43^	[39]
${\mathrm{Ca}}^{2+}+G^{-2}\;⇄$ CaG	K _sp(289.15K)_ = 10^1.41^	[45]
Ca^2+^ + H_2_PO_4_^−^ ⇄ CaH_2_PO_4_^+^	K _sp(289.15K)_ = 31.9	[42]
${\mathrm{Ca}}^{2+}+{{\mathrm{HPO}}_{4}}^{2-}\;⇄$ CaHPO_4_	K _sp(289.15K)_ = 6.81 × 10^2^	[42]
Ca^2+^ + PO_4_^3−^ ⇄ CaPO_4_^−^	K _sp(289.15K)_ = 3.46 × 10^6^	[42]

**Table 3:** Concentrations of all species involved in the chemical reactions for the synthesis of samples A, B, C, D, E, and F.

	Sample A	Sample B	Sample C	Sample D	Sample E	Sample F
Ca(OH)_2_	0	1.334 × 10^−12^	0	2.711 × 10^−10^	0	3.000 × 10^−8^
Ca(OH)^+^	1.468 × 10^−8^	0.00245	0.10243	0.04228	1.476 × 10^−6^	0.44035
Ca^2+^	0.88603	17.42041	0.10243	24.26746	0.90356	23.53375
OH^−^	1.279 × 10^−7^	0.00277	1.290 × 10^−6^	0.03108	4.068 × 10^−5^	0.35424
H_3_PO_4_	0.01057	2.415 × 10^−8^	0.00593	2.270 × 10^−11^	4.596 × 10^−6^	1.960 × 10^−14^
H_2_PO_4_^−^	0.92035	0.04786	5.42343	0.00050	0.13323	4.948 × 10^−6^
HPO_4_^2−^	5.550 × 10^−4^	0.59145	0.03285	0.06707	0.02703	0.00762
PO_4_^3−^	1.747 × 10^−12^	3.443 × 10^−5^	9.980 × 10^−10^	3.906 × 10^−5^	2.900 × 10^−8^	5.423 × 10^−5^
H_2_G	0.00035	0.00054	0.90892	2.790 × 10^−5^	0.00321	-
HG^−^	0.36869	12.96555	9.90568	7.39003	1.11383	-
G^2−^	1.193 × 10^−6^	0.86074	0.00032	5.22065	0.00121	-
CaHG^+^	14.37115	12.24621	48.59966	10.76048	43.29262	-
CaH_2_PO_4_^+^	42.51825	0.05358	31.53652	0.00087	6.13747	7.812 × 10^−6^
CaHPO_4_	0.89393	28.47270	7.25726	5.44717	42.48979	0.54058
CaPO_4_^−^	2.339 × 10^−8^	16.96805	0.00199	36.33896	0.37035	41.05891
CaG	7.261 × 10^−5^	1.56401	0.00268	16.18347	0.07203	-
H^+^	0.06407	2.534 × 10^−6^	0.00585	2.152 × 10^−7^	0.00019	2.025 × 10^−8^
pH calculation	4.19	8.60	5.23	9.67	6.71	10.69

## Data Availability

Not applicable.
